# Inhibition of ubiquitin‐specific protease 2 causes accumulation of reactive oxygen species, mitochondria dysfunction, and intracellular ATP decrement in C2C12 myoblasts

**DOI:** 10.14814/phy2.14193

**Published:** 2019-07-28

**Authors:** Mayuko Hashimoto, Natsuko Saito, Haru Ohta, Kumiko Yamamoto, Asuka Tashiro, Kosuke Nakazawa, Osamu Inanami, Hiroshi Kitamura

**Affiliations:** ^1^ Laboratory of Veterinary Physiology, School of Veterinary Medicine Rakuno Gakuen University Ebetsu Japan; ^2^ Laboratory of Radiation Biology, Graduate School of Veterinary Medicine Hokkaido University Sapporo Japan

**Keywords:** USP, myoblast, mitochondria, respiratory chain, oxidative phosphorylation

## Abstract

Ubiquitin‐specific protease 2 (USP2) is considered to participate in the differentiation of myoblasts to myotubes, however, its functions in myoblasts under growth conditions remain elusive. In this study, we analyzed the physiological roles of USP2 in myoblasts using *Usp2* knockout (KO) C2C12 cells as well as a USP2 specific inhibitor. In addition to the disruption of differentiation, clustered regularly interspaced short palindromic repeats/Cas9‐generated *Usp2*KO cells exhibited inhibition of proliferation compared to parental C2C12 cells. *Usp2*KO cells reduced the accumulation of intracellular adenosine triphosphate (ATP) content and oxygen consumption. Moreover, *Usp2*KO cells had fragmented mitochondria, suggesting that mitochondrial respiration was inactive. The deficiency of *Usp2* did not affect the enzymatic activities of respiratory chain complexes I, III, IV, and V. However, mitochondrial membrane permeability—evaluated using calcein AM‐cobalt staining—was increased in *Usp2*KO cells. The membrane potential of *Usp2*KO cells was clearly decreased. *Usp2*KO cells accumulated reactive oxygen species (ROS) in the mitochondria. The USP2‐selective inhibitor ML364 also increased the levels of mitochondrial ROS, and modulated the membrane potential and morphology of the mitochondria. These effects were followed by a decrement in the intracellular content of ATP. Based on these findings, we speculate that USP2 may be involved in maintaining the integrity of the mitochondrial membrane. This process ensures the supply of ATP in myoblasts, presumably leading to proliferation and differentiation.

## Introduction

In addition to motor function, skeletal muscle plays a pivotal role in the maintenance of energy homeostasis. Skeletal muscle consumes more than 70% of the glucose present in the body (DeFronzo, 1988) and secretes metabolism‐regulating myokines (Whitham and Febbraio, 2016). In the differentiation process of the myocyte lineage, satellite cells undergo differentiation into myoblasts and subsequently differentiate into myofibers (Yin et al., 2013). Accumulating evidence indicates that defects in myofibers and their progenitor cells cause muscular diseases such as sarcopenia and muscle dystrophy (Always et al., 2014; Dumont et al., 2015). Another study, using a mouse model, suggested that muscle progenitor cells with damaged mitochondria induce sarcopenia (Wang et al., 2013; García‐Prat et al., 2016). A sufficient supply of ATP in muscle progenitor cells, including satellite cells and myoblasts, is thus likely to be critical in maintaining the functional integrity of skeletal muscle.

A wide variety of cells switch their metabolic profile between glycolysis dominance and oxidative phosphorylation (OXPHOS) dominance depending on their states (Kitamura et al., 2008; Escoll and Buchrieser, 2018; Ravera et al., 2018; Waters et al., 2018). Although myofibers preferentially obtain ATP by OXPHOS, satellite cells and myoblasts may acquire ATP predominantly by glycolysis (Leary et al., 1998; Sin et al., 2016; Wagatsuma and Sakuma, 2013). On the other hand, a previous study demonstrated that the differentiation and proliferation of myoblasts is highly dependent on both OXPHOS and glycolysis (Riddle et al., 2018). Therefore, activated myoblasts seem to utilize both OXPHOS and glycolysis for ATP supply. OXPHOS was found to be carried out by the respiratory chain complex V located in the inner membrane of the mitochondrium (Dudkina et al., 2010). This ~600 kDa complex of F_1_F_0_ ATP synthase produces ATP by taking advantage of the proton gradient generated by complexes I, III, and IV (Dudkina et al., 2010). The retention of the proton gradient between the matrix and inner membrane spaces of the mitochondrium is thus necessary for complex V‐driven OXPHOS.

Ubiquitin, which is a small protein comprised of 76 amino acids, binds to target proteins followed by protein digestion or protein–protein interaction (Walczak et al., 2012). Protein ubiquitination, which is a reversible posttranslational process, is regulated by ubiquitin ligases and deubiquitinases (Lill and Wertz, 2014). The ubiquitin‐specific proteases (USP) are the largest family of ubiquitin proteases, consisting of ~50 members in mammalian species (Reyes‐Turcu et al., 2009). USP2, a member of the USP family, is widely expressed in organs, with relatively higher expression levels reported in the testis and skeletal muscle (Gousseva and Baker, 2003; Kitamura et al., 2013). It has diverse functions in a wide variety of cells, such as pro‐ and antiapoptotic effects in cancerous cells (Gewies and Grimm, 2003; Priolo et al., 2006), regulatory roles in cytokine production in macrophages (Zhang et al., 2014; Sun et al., 2016; Kitamura et al., 2017), and modulation of glucose and lipid metabolism (Molusky et al., 2012; Kitamura et al., 2013; Nelson et al., 2016; Saito et al., 2017). In a previous study, dominant negative forms of USP2 were found to interrupt the differentiation of L6 myoblasts into myofibers indicating the involvement of USP2 in myocyte differentiation (Park et al., 2002). Despite this existing evidence, the detailed physiological roles of USP2 in myoblasts remain elusive. In this study, we investigated the roles of USP2 in myoblasts, focusing on energy metabolism, by using *Usp2* gene knockout (KO) cells.

## Materials and Methods

### Cell culture

The mouse myoblast cell line C2C12 was obtained from RIKEN Bioresource Center (Tsukuba, Japan). The cells were grown in Dulbecco’s modified Eagle medium (DMEM) containing 4.5 g/L glucose and 10% fetal calf serum (FCS). The cells underwent differentiation into myotubes for 7 days in DMEM containing 4.5 g/L glucose and 2% horse serum. In some experiments, we treated the cells with the USP2‐selective inhibitor ML364 (10 µmol/L) (MedChemExpress, Monmouth Junction, NJ, USA) or vehicle (DMSO, 2 mmol/L) for 0.5, 2, 4, 8, 12 h, or 5 days.

### Generation of *Usp2*KO C2C12 cells

*Usp2* KO C2C12 cells were generated using the clustered regularly interspaced short palindromic repeats (CRISPR)/Cas9 method (Sander and Joung, 2014). A pair of oligonucleotides was designed for the region coding isopeptidase as follows: 5′‐CACCGCTGGCTGGTCTTCGAAACCT‐3′; 5′‐AACAGGTTTCGAAGACCAGCCAGC‐3′ (corresponding to 44,089,153 to 44,089,173 of mouse genome, version GRCm38.p2; Sigma Aldrich, St. Louis, MI, USA). After annealing, the double‐stranded oligonucleotides were ligated into *Bbs*I‐digested pSPCas9(BB)‐2A‐GFP (Addgene, Cambridge, MA, USA) using T4 ligase (New England Biolabs, Ipswich, MA, USA). The resultant plasmid was termed pSP‐*Usp2*gRNA‐Cas9‐2A‐GFP.

**Figure 1:**
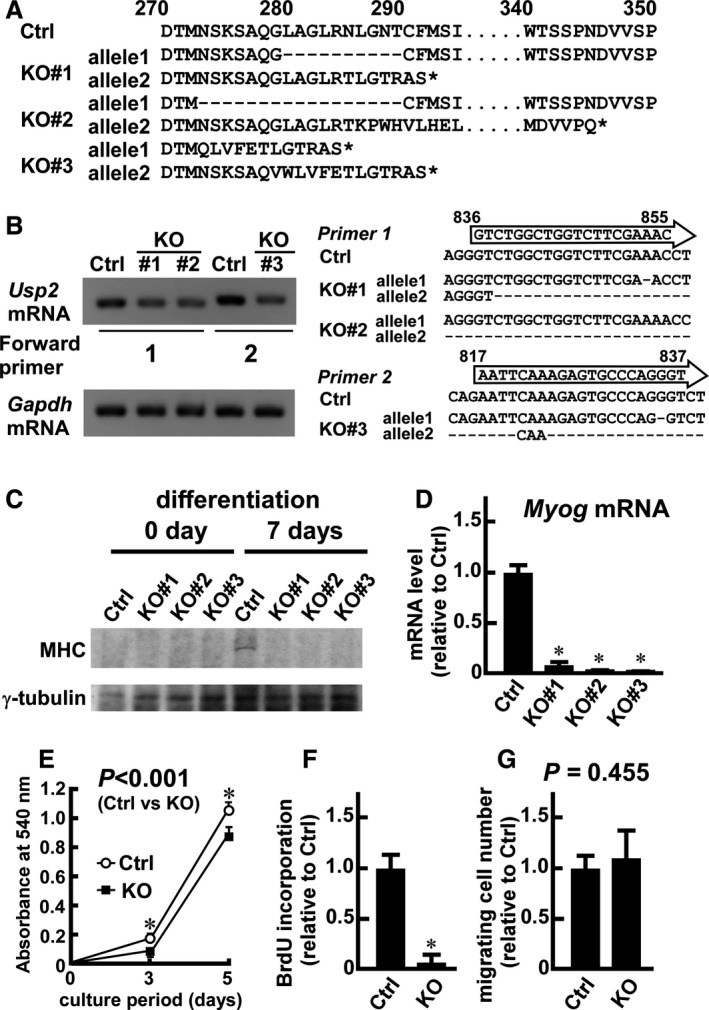
Generation and characterization of *Usp2* knockout (KO) C2C12 cells. Taking advantage of the clustered regularly interspaced short palindromic repeats/Cas9 method, we established three independent *Usp2*KO cell lines. (A) Alignment of putative amino sequences of *Usp2* gene products in gene‐engineered (KO#1, KO#2, and KO#3) and control C2C12 cells. The amino acid numbers from the N‐terminal are shown. Dashes and asterisks represent deletion and stop codons, respectively. (B) RT‐PCR analysis of the gene‐engineered regions of *Usp2* mRNA using primer 1 or primer 2 as forward primers. Left, Representative images of three experiments. As a reference, *Gapdh* mRNA was also amplified. Right, cDNA sequences of the mutated region of control and *Usp2*KO C2C12 cells. The nucleotide numbers from the initial codon are shown. The location of the PCR primers is also depicted as arrows. Dashes represent deletion. (C) Expression of the myosin heavy chain (MHC) in *Usp2*KO and control C2C12 cells before and after differentiation. Representative western blot images of three experiments are shown. As a loading control, we also detected γ‐tubulin. (D) Myogenin (*Myog*) mRNA level in *Usp2*KO and control cells. After incubation in the differentiation medium for 7 days, the cells were subjected to qRT‐PCR analysis. Expression values of *Myog* mRNA were normalized to the 18s rRNA levels and are relative to those detected in control C2C12 cells. (E) Growth curves of *Usp2*KO and control cells. After seeding 500 cells in a 96‐well plate, cell numbers were measured by the MTT methods on days 0, 3, and 5. (F) Bromodeoxyuridine (BrdU) incorporation analysis in *Usp2*KO and control cells. Cells (500 cells) were seeded in a 96‐well plate, incubated in the presence of BrdU for 5 days and subjected to enzyme‐linked immunosorbent assay (ELISA) to measure the amount of incorporated BrdU. (G) Quantification of migration of *Usp2*KO and control cells. Two days after seeding, migrated cells were monitored. Data are means ± one standard deviation of five (D, F), six (E), or three (G) wells per treatment group in a representative experiment for three experimental series. *P < 0.05 versus control cells (E) or *Usp2*KO cells (F). The *P*‐values are also shown (E, G).

Transfection of pSP‐*Usp2*gRNA‐Cas9‐2A‐GFP into C2C12 cells was performed using Lipofectamine 2000 (Thermo Fisher Scientific). Fluorescent cells were sorted using a fluorescence‐activated cell sorting (FACSAria II cell sorter;BD Bioscience, Franklin Lakes, NJ, USA) assay 2 days after transfection. Subsequently, the sorted cells were seeded on a dish and colonized clones were picked using cloning discs (Sigma Aldrich). Genomic DNA from the obtained clones was isolated using the GeneElute Mammalian Genomic DNA Miniprep kit (Sigma Aldrich). Subsequently, *Usp2* gene fragments near the edited region were amplified using polymerase chain reaction (PCR; corresponding to 44,089,969–44,089,488 bp of the mouse genome), ligated into a pGEM plasmid (Promega, Madison, WI, USA), and cloned in an *Escherichia coli* DH5α strain. Four or more *E. coli* clones for each *Usp2*KO cell line were subjected to DNA sequencing, which was performed by Hokkaido System Science (Sapporo, Japan). Eventually, we obtained three independent *Usp2* KO cells with different mutations in the *Usp2* locus (Fig. 1A).

### RNA extraction and reverse transcription‐PCR (RT‐PCR)

Total RNA was extracted using RNAiso Plus (Takara Bio, Otsu, Japan), and was subjected to reverse transcription with random primers and M‐MLV reverse transcriptase (Wako, Osaka, Japan) according to the instructions provided by the manufacturer. Subsequently, RT‐PCR for the *Usp2* gene was performed with GoTaq hot start polymerase (Promega) using primers 5′‐GTCTGGCTGGTCTTCGAAAC‐3′ (primer 1, Fig. 1B) and 5′‐CTTCCATGAGGGCCGTGT‐3′ for the *Usp2* transcript of control C2C12 cells and KO#1 and KO#2 cells; 5′‐ AATTCAAAGAGTGCCCAGGGT‐3′ (primer 2) and 5′‐CTTCCATGAGGGCCGTGT‐3′ for the *Usp2* transcript of control C2C12 cells and KO#3 cells; 5′‐GGTGCTGAGTATGTCGTGGA‐3′ and 5′‐GTGGTTCACACCCATCACAA‐3′ for the transcript of glyceraldehyde 3‐phosphate dehydrogenase (*GAPDH*). After pretreatment at 98°C for 2 min, the reaction (35 cycles) was performed as follows: 95°C for 30 sec, 59°C for 30 sec, and 72°C for 30 sec. The amplified fragment was separated through electrophoresis using a 3% agarose gel (Agarose 21, Nippongene, Toyama, Japan). The gel was stained with 500 ng/mL of ethidium bromide, and scanned using an AX‐310B gel imager (Bio Craft, Tokyo, Japan). Digitalization of images was performed using ShowBiz software (ArcSoft, Fremont, CA, USA).

### Western blotting analysis

Western blotting analysis was performed as previously described (Saito et al., 2017). Briefly, total cellular protein was extracted with RIPA buffer (50 mmol/L Tris‐HCl [pH 7.6], 150 mmol/L sodium chloride, 1% Nonidet P‐40, 0.5% sodium deoxycholate, 0.1% SDS, 1 mmol/L EDTA, protease inhibitor cocktail [Roche Life Science, Basel, Switzerland]). After a twofold dilution with a loading buffer (100 mmol/L Tris‐HCl [pH 6.8], 4% SDS, 20% glycerol, 0.01% bromophenol blue), the protein samples were denatured at 100°C for 5 min, and loaded in a 10% sodiumdodecyl sulfate‐polyacrylamide gel. Samples in the gel were then transferred onto a PVDF membrane (Merck Millipore, Burlington, MA, USA), and blocked with Blocking One reagent (Nacalai, Kyoto, Japan) at room temperature for 1 h. The membranes were immersed in HIKARI enhancer solution (Nacalai) containing anti‐USP2 (AP2131b and AP2131c; Abgent, San Diego, CA), anti‐human myosin heavy chain goat antibody (sc‐376157; Santa Cruz Biotechnology, Dallas, TX, USA) or anti‐human γ‐tubulin rabbit antibody (ab11317; Abcam, Cambridge, UK) at 4°C overnight. The membranes were allowed to react with horseradish peroxidase‐conjugated anti‐goat IgG (ab6789, Abcam) or anti‐rabbit IgG (#7074S; Cell Signaling Technology, Danvers, MA, USA) at room temperature for 3 h. After washing with Tris‐buffered saline (pH 7.6) with 0.05% Tween 20, immunological signals were visualized with a Chemi Lumi One Super Kit (Nacalai) and monitored using the EZ‐capture system (Atto, Tokyo, Japan).

### Quantitative reverse transcription‐polymerase chain reaction (qRT‐PCR)

Quantitative PCR (qPCR) was performed with the KAPA SYBR Green Fast qPCR kit (KAPA Bioscience, Wilmington, CA, USA) using an ECO qPCR system (Illumina, San Diego, CA, USA). The primers used for the qPCR were as follows: 5′‐TACCCAAGGTGGAGATCCTG‐3′ and 5′‐GCATCTGAGTCGCCACTGTA‐3′ for myogenin (official gene symbol *Myog*); 5′‐TCGTCTTCGAAACTCCGACT‐3′ and 5′‐CGCGGTTCTATTTTGTTGTT‐3′ for 18s ribosomal RNA (official gene symbol *Rn18n*).

### Cell proliferation

Cell proliferation was evaluated with two different assays, namely 3‐(4, 5‐dimethylthial‐2‐yl)‐2, 5‐diphenyltetrazalium bromide (MTT) assay and bromodeoxyuridine (BrdU) incorporation assay. In the MTT assays, the cell number was estimated by the amount of formazan catalyzed by mitochondrial reductase. The MTT assays were performed as previously described (Ishino et al., 2018). Briefly, the cells were seeded at 5 × 10^2^/well. After culturing for 0, 3, and 5 days, the cells were incubated in the presence of MTT (0.5 mg/mL, Nacalai) for 4 h. Formazan was extracted using the same volume of an extraction buffer (5 mmol/L HCl, 5% sodium lauryl sulfate), and measured at 540 nm using an iMark microplate reader (BioRad, Hercules, CA, USA). Under this condition, *Usp2*KO and control C2C12 cells did not reach confluency even after 5 days of culture. The incorporation of BrdU in proliferative cells was measured using a BrdU Cell Proliferation kit (Roche Diagnostics, Mannheim, Germany) according to the manufacturer’s manual. The chemiluminescent signal was measured using an AB‐2300 (Atto).

### Cell migration

Cell migration was analyzed using an Oris Cell Migration kit (Platypus Technologies, Madison, MI, USA) according to the manufacturer’s manual. Briefly, *Usp2*KO and control C2C12 cells were seeded on a plate supplied with the kit. Two days after seeding, the nuclei of the cells were stained with Hoechst33342 (5 µg/mL, Thermo Fisher Scientific) and subsequently monitored using a laser‐scanning confocal microscope C2 (Nikon, Tokyo, Japan). The fluorescence intensity was digitalized using NIS‐Elements Advance Research software (Nikon) and further analyzed with ImageJ software (Schneider et al., 2012).

### Intracellular ATP content

Intracellular ATP content was measured using an ATP measurement solution (Toyo B‐Net, Tokyo, Japan). After seeding in a plate (1 × 10^3^ cells/well), the cells were incubated in glucose‐containing DMEM supplemented with 10% FCS for 12 h. Subsequently, the medium was changed to glucose‐containing or glucose‐free DMEM supplemented with 10% FCS, and the cells were further incubated for 12 h. Then, twofold concentrated ATP measurement solution was added to the medium and mixed vigorously using a plate shaker for 1 min. After 10 min, the chemiluminescent signal was measured using a VICTOR Nivo multimode microplate reader (Perkin Elmer, Waltham, MA, USA).

### Oxygen consumption

Oxygen consumption was measured by electron spin resonance (ESR) as previously described (Yamamori et al., 2012). Lithium 5,9,14,18,23,27,32.36‐octa‐*n*‐butoxy‐2,3‐naphthalocyanine (LiNc‐BuO) was synthesized according to the method previously described (Pandian et al., 2003). Trypsinized cells suspended in serum‐free DMEM containing 1 mg/mL LiNc‐Buo and 2% dextran were packed in a glass capillary tube. ESR measurements were performed using a JEOL‐RE X‐band spectrometer (JEOL, Tokyo, Japan) with a cylindrical TE011 mode cavity (JEOL). The cavity was maintained at 37°C using a temperature controller (ES‐DVT3; JEOL). The spectrometer conditions were as follows: incident microwave power, 1 mW; field modulation amplitude, 6.3 µT; scan range, 0.5 mT. Spectral line widths were assessed using a Win‐Rad radical analyzer system (Radical Research, Tokyo, Japan) and converted to partial pressure of oxygen (*p*O_2_) values as previously described (Yamamoto et al., 2018).

### Visualization of mitochondria

Mitochondria were stained with 250 nmol/L MitoTracker Red CMXRos (Thermo Fisher Scientific), or 200 nmol/L MitoTracker Green FM (Thermo Fisher Scientific) for 30 min. Subsequently, the cells were fixed with 4% paraformaldehyde for 10 min. In some experiments, we transfected a plasmid encoding the mitochondria‐targeting mCherry (kindly provided by Dr. T. Yamamori, Hokkaido University) (Bo et al., 2018) to *Usp2*KO and control C2C12 cells using Lipofectamine 2000 (Thermo Fisher Scientific). After staining the nuclei with Hoechst33342, the cells were monitored using a laser‐scanning confocal microscope C2. Series of 16 bit‐images were captured at 0.25 µmol/L intervals from the bottom to the top of the cells.

Three‐dimensional images of mitochondria were reconstituted using ImageJ. Mitochondrial mass was calculated using the Sync Measure 3D function of ImageJ. The threshold signal intensity was the sum of the mean intensity of the cell‐absent area plus two standard deviations.

To evaluate the mitochondrial classification, a series of images were integrated using the Z Projection function of ImageJ. Fifty mitochondria were randomly selected from every specimen. Mitochondria with a major axis>3 µmol/L were recognized as elongated and network‐forming mitochondria.

### Mitochondrial DNA content (mtDNA)

Total DNA was extracted with Universe ALL Extraction Buffer II (Yeastern Biotech, Taipei, China). The mtDNA content was evaluated by qPCR. The D‐loop region of the mtDNA was amplified using the primers 5′‐GCCCATTAAACTTGGGGGTA‐3′ and 5′‐TTATGTTGGTCATGGGCTGA‐3′ as previously described (Yamamori et al., 2012). A 106‐bp element of the *Usp2* gene, corresponding to 44,090,048–44,090,153 bp of the mouse genome (version GRCm38.p2), was also amplified as a reference using the primers 5′‐CTTCAGTGCCTGAGCAACAC‐3′ and 5′‐ CTTCCATGAGGGCCGTGT‐3′. The amplified region was distinct from the gene‐edited region (44,089,132–44,089,182 bp of the mouse genome).

### Isolation of mitochondria

Trypsinized cells (5 × 10^6^) were suspended in a hypotonic buffer (10 mmol/L Tris HCl [pH 7.5], 10 mmol/L NaCl, 1.5 mmol/L MgCl_2_) at 4°C for 10 min, and then homogenized using a Dounce homogenizer with 35 strokes. After addition of 2/3 volume of the mannitol–sucrose solution (12.5 mmol/L Tris‐HCl [pH 7.5], 525 mmol/L mannitol, 175 mmol/L sucrose, 2.5 mmol/L EDTA), the homogenate was centrifuged (17,000 × *g*) at 4°C for 15 min. The mitochondria pellet was suspended in a buffer containing 100 mmol/L Tris‐HCl (pH 7.5), 100 mmol/L sucrose, 10 mmol/L EDTA, 46 mmol/L KCl, and 0.5% bovine serum albumin (BSA), subjected to measurement of protein concentration, and stored at − 80°C until use.

### Activities of respiratory chain complexes

The enzymatic activities of mitochondrial respiratory chain complexes were measured by colorimetric determination assays using a SpectraMax Paradigm (Molecular Devices, San Jose, CA) at 30°C. As previously reported (Tatarkova et al., 2011), the molar absorptivity of NADH and cytochrome *c* was estimated as 6.2 mmol/L·cm and 19.6 mmol/L·cm, respectively. The concentrations of NADH and cytochrome *c* were calculated according to the Lambert–Beer law. The activity of each mitochondrial complex was measured using mitochondria frozen and thawed once, and defined as change in the amount of substrates in relation to the mitochondrial protein content and reaction period. The activity of complexes with the exception of Complex IV was determined by subtracting the values of the respective inhibitor‐treated samples.

Complex I activity was measured by the rotenone‐inhibitable oxidation of NADH. Isolated mitochondria (50 µg/mL of protein) were preincubated with 25 mmol/L KH_2_PO_4_ (pH 7.2), 5 mmol/L MgCl_2_, 2.5 mg/mL BSA, 2 mmol/L KCN, 65 µmol/L coenzyme Q1 (Cayman Chemical, Ann Arbor, MI, USA), and 2 µg/mL antimycin A (Santa Cruz Biotechnology) for 5 min. Subsequently, rotenone (2 µg/mL, Sigma Aldrich) or vehicle with NADH (325 µmol/L, Nacalai) were added. Absorbance at 340 nm was measured every 30 sec for 20 min after starting the reaction.

Complex III activity was measured by the antimycin A‐inhibitable reduction of oxidative cytochrome c (III). The mitochondria were preincubated with 50 mmol/L KH_2_PO_4_ (pH 7.2), 0.1% BSA, 0.1 mmol/L EDTA, 2 mmol/L KCN, and 35 µmol/L coenzyme Q1 in the presence or absence of antimycin A (2 µg/mL) for 5 min. Subsequently, 60 µmol/L oxidative cytochrome c (Sigma Aldrich) was applied as a substrate. Absorbance at 550 nm was measured every 30 sec for 20 min after starting the reaction.

Complex IV activity was measured by the oxidization of reduced cytochrome *c* (II). Immediately before an experiment, cytochrome *c* (II) was reduced by incubation with 0.5 mmol/L DTT for 15 min. The mitochondria were preincubated with 25 mmol/L KH_2_PO_4_ (pH 7.2) and 0.45 mmol/L n‐dodecyl‐β‐D‐maltoside for 5 min, and then added 15 µmol/L reduced cytochrome *c* (II). Absorbance at 550 nm was measured every 30 sec for 20 min after starting the reaction.

Complex V/F_1_F_0_ ATPase phosphorylates ADP to form ATP using a proton gradient under physiological conditions. On the other hand, the enzyme also catalyzes the dephosphorylation of ATP to ADP and phosphate in the presence of rotenone (Kipp and Ramirez, 2001). Thus, the activity of Complex V was measured through the oligomycin‐inhibitable production of ADP in the presence of rotenone as previously reported with modifications (Kipp and Ramirez, 2001). ADP, which is dephosphorylated by Complex V in proton gradient‐collapsed mitochondria, drives the consecutive reaction of pyruvate kinase and lactate dehydrogenase (LDH) resulting in decrement of NADH (Absorbance, 340 nm). The frozen‐thawed mitochondrial solution was preincubated with 50 mmol/L HEPES, 3 mmol/L MgCl_2_, 50 mmol/L KCl, 0.2 mmol/L EDTA, 2 mmol/L phosphoenolpyruvic acid, 100 µmol/L rotenone, 10 U/mL lactate dehydrogenase (Oriental Yeast, Tokyo, Japan), and 10 U/mL pyruvate kinase (MP Biomedicals, Santa Ana, CA, USA) for 5 min. Subsequently, 2 mmol/L ATP and 0.5 mmol/L NADH were added in combination with either oligomycin (10 µg/mL, Wako) or vehicle. Absorbance at 340 nm was measured every 30 sec for 20 min after starting the reaction.

### Mitochondrial membrane permeability

Mitochondrial membrane permeability was evaluated by the calcein‐AM‐cobalt method (Petronilli et al., 1998). The cells were treated with 1 µmol/L of calcein‐AM (Sigma Aldrich) for 30 min. After washing with phosphate‐buffered saline (PBS), the cells were incubated in the presence of 1 mmol/L cobalt chloride for 30 min. Subsequently, the cells were subjected to monitoring of fluorescent signals derived from calcein‐AM retained in the mitochondria using a FACS Verse (BD Biosciences, San Jose, CA, USA).

### Mitochondrial membrane potential

The mitochondrial membrane potential was assessed by the stainability of the cationic dye tetramethylrhodamine methyl ester (TMRM; Setaresh Biotech, Eugene, OR, USA), which is concentrated in depolarized mitochondria releasing an orange–red fluorescent signal. After treatment with 20 nmol/L of TMRM for 30 min, the cells were subjected to analysis using a FACS Verse.

### Mitochondrial reactive oxygen species (ROS) production

The production of mitochondrial ROS was measured using the MitoSOX Red superoxide indicator (Thermo Fisher Scientific). The cells were treated with 5 µmol/L MitoSOX Red reagent for 30 min. After washing with warm PBS, the cells were monitored using a FACS Verse or an IX71 fluorescence microscope (Olympus, Tokyo, Japan) connected to a DP73 cooling CCD camera (Olympus).

### LDH assay

The toxicity of ML364 was evaluated using a LDH cytotoxicity assay kit purchased from Dojindo (Kumamoto, Japan). Assays were performed according to the instructions provided by the manufacturer.

### Statistical analysis

**Figure 2:**
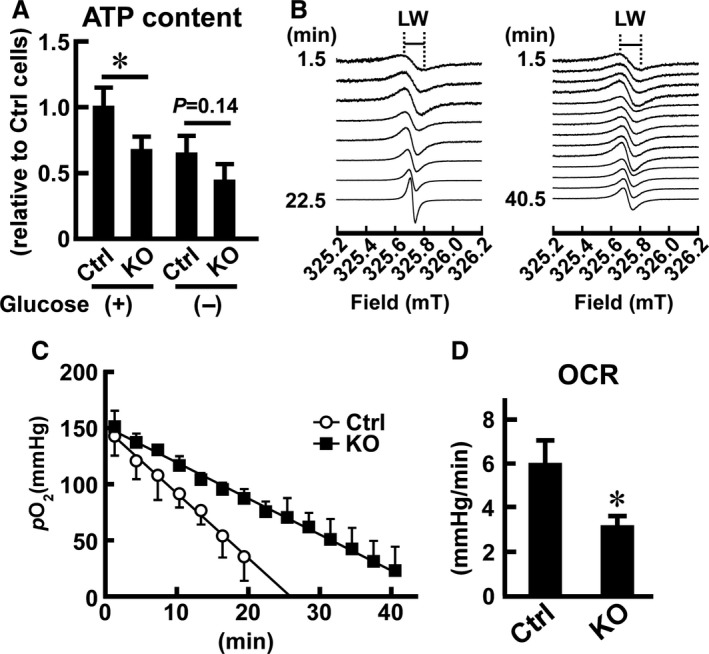
Effects of *Usp2* deficiency on intracellular ATP content and oxygen consumption in C2C12 cells. (A) Intracellular content of ATP in *Usp2*KO (KO) and control (Ctrl) C2C12 cells cultured in normal (+) and glucose‐free (−) DMEM supplemented with 10% FCS for 12 h. Values were calculated as ATP content per single cell and are represented as relative to those detected in control cells. (B) Representative ESR spectra obtained from control (left) or *Usp2*KO (right) cells. LW stands for line width. (C) Time course of changes in *p*O2 in control (open circle) and *Usp2*KO (closed square) cells. (D) Oxygen consumption rates in *Usp2*KO and control cells. Data are expressed as means ± one standard deviation of four wells in a representative experiment of three experimental series (A) and three experiments (C, D). *P < 0.05 versus control cells (A, D). *P‐*values obtained from Tukey’s test are also shown (A).

**Figure 3:**
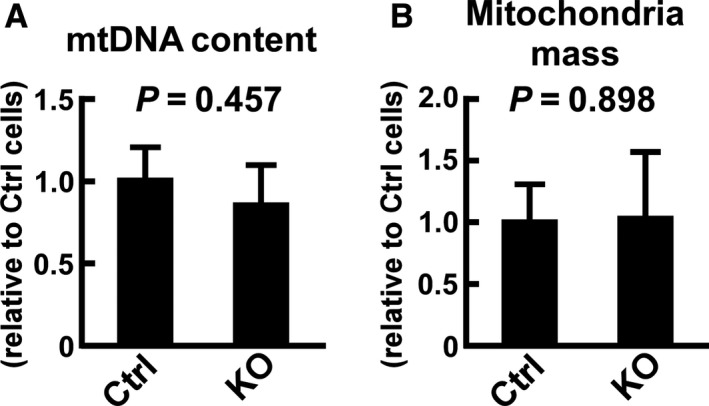
Effects of *Usp2* deficiency on the mitochondrial content in C2C12 cells. (A) The mitochondrial DNA (mtDNA) content of *Usp2*KO (KO) and control (Ctrl) cells assessed by quantitative PCR. To measure the mtDNA content, the D‐loop encoded in the mtDNA was amplified. Values were normalized to genomic DNA content evaluated by the copy number of the *Usp2* gene. (B) Total mitochondrial mass. The total mitochondrial mass was calculated based on the sequence images of MitoTracker Red CMXRos‐stained cells. Data are expressed as means ± one standard deviation of three experiments (A) and 11–13 cells in three wells in a representative experiment of three experimental series (B). *P* values obtained from Student’s *t*‐test are also shown.

**Figure 4:**
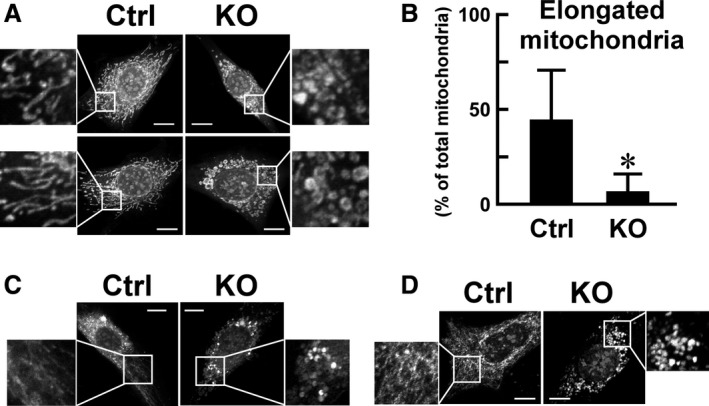
Morphology of the mitochondria of *Usp2*KO and control C2C12 cells. Mitochondria were stained with MitoTracker Red CMXRos (A, B), MitoTracker Green FM (C), and mitochondria‐targeting mCherry (D). Nuclei were stained with Hoechst33342. (A, C, D) Representative images of control C2C12 cells (Ctrl, left) and *Usp2*KO cells (KO, right) for three experiments. Enlarged views of square areas are also shown to indicate mitochondrial morphology. Scale bars represent 10 µm. (B) Proportion of elongated mitochondria among total mitochondria. A major axis>3 µm was indicative of elongated mitochondria. Data are expressed as means ± one standard deviation of 11 (*Usp2*KO) and 13 (control) cells of three wells of a representative experiment of three experimental series.*P < 0.05 versus control cells.

**Figure 5:**
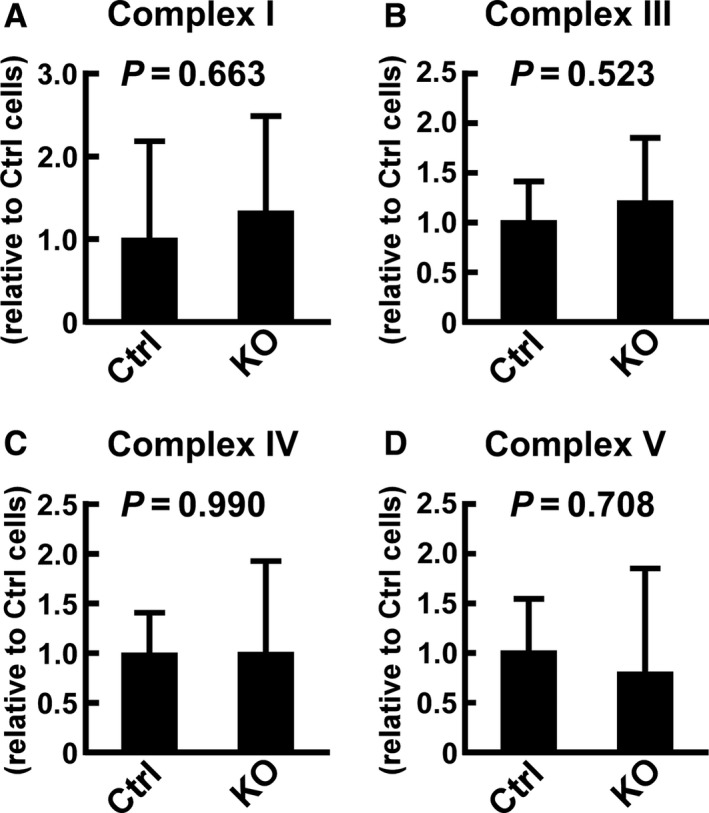
Effects of *Usp2* deficiency on the enzymatic activities of respiratory chain complexes in C2C12 cells. Mitochondria isolated from *Usp2*KO (KO) and control (Ctrl) C2C12 were subjected to colorimetric assays of Complexes I (A), III (B), IV(C), and V(D). For Complexes I, III, and V, we calculated the net activity by subtracting the background activity of the cells treated with respective inhibitors (2 µg/mL rotenone, 2 µg/mL antimycin A, and 10 µg/mL oligomycin for Complex I, III, and V, respectively). Values were represented as relative to those observed in control cells. Data are expressed as means ± one standard deviation of five experiments. *P* values obtained from Student’s *t*‐test are also shown.

**Figure 6:**
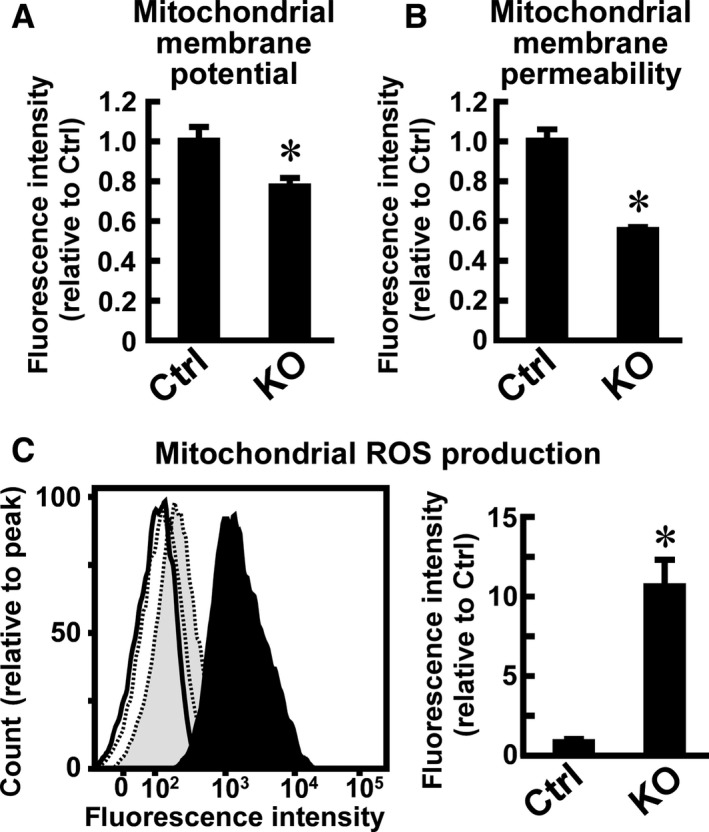
Effects of *Usp2* deficiency on mitochondrial membrane potential, membrane permeability, and reactive oxygen species (ROS) production in C2C12 cells. *Usp2*KO (KO) and control (Ctrl) C2C12 cells were cultured in the presence of 20 nmol/L TMRM (A), 1 µmol/L calcein AM and 1 mmol/L cobalt chloride (B), or 5 µmol/L MitoSox Red (C) for 30 min to detect the membrane potential, membrane permeability, or ROS production of mitochondria, respectively. The fluorescence intensity of cells was analyzed using a flow cytometer. After subtraction of the fluorescence intensity of nonstained cells, the median fluorescent signal values were calculated. Data are expressed as means ± one standard deviation of three (A, B) and four (C, right) experiments. A representative fluorescence‐activated cell sorting (FACS) assay image of mitochondrial ROS detection is also shown (left panel of C). Solid and dotted lines represent *Usp2*KO and control cells, respectively. Filled and air plots represent MitoSox Red‐stained and unstained cells, respectively.

**Figure 7:**
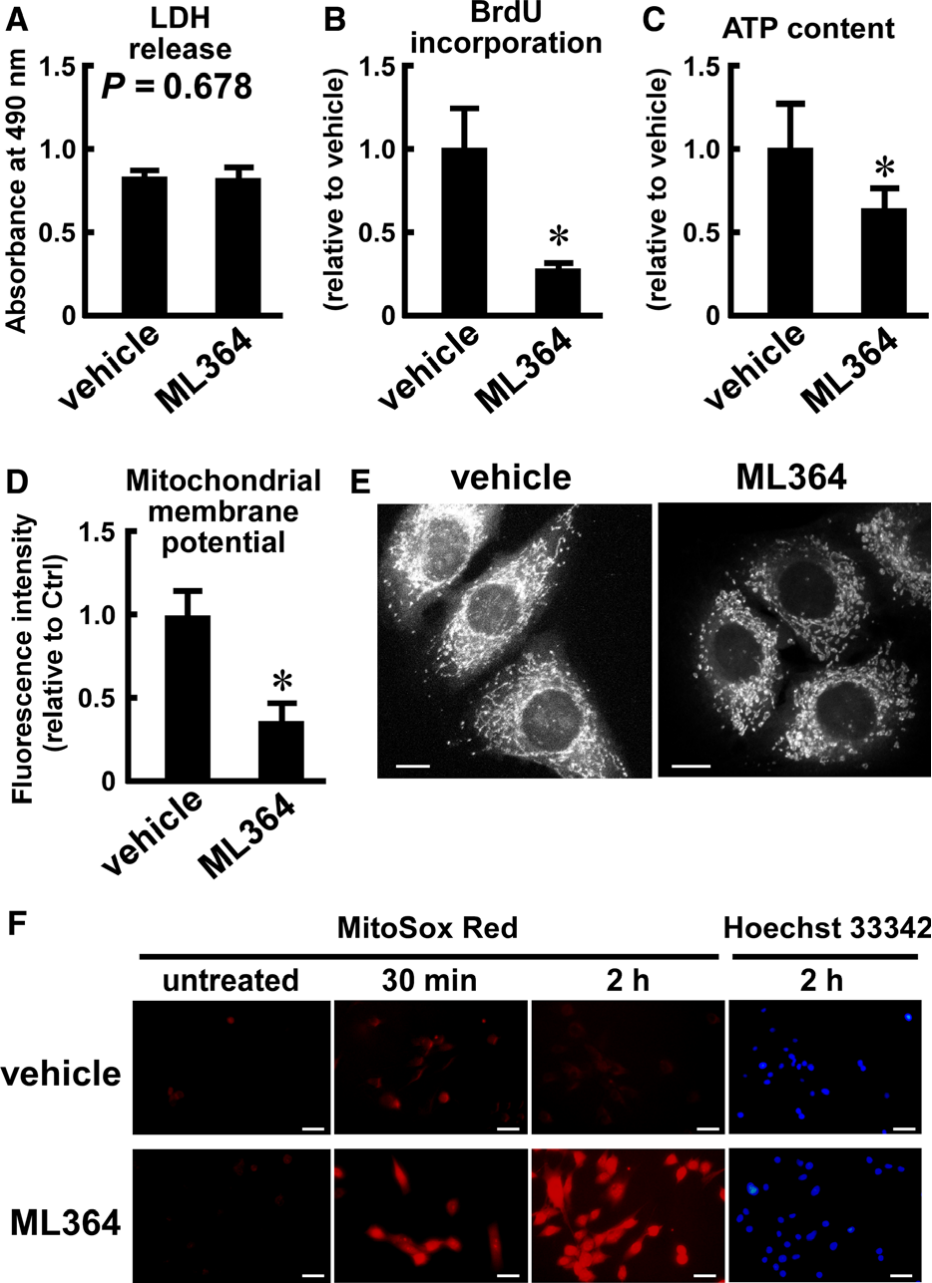
Effects of ML364 on cytotoxicity, proliferation, intracellular level of ATP, mitochondrial membrane potential, mitochondria morphology, and accumulation of ROS in C2C12 cells. C2C12 cells were treated with ML364 (10 µmol/L) or vehicle (DMSO) for 12 h (A), 5 days (B), 8 h (C, D), 4 h (E), or 0.5–2 h (F). (A) LDH content in the culture medium. (B) BrdU incorporation assay. Cells (500 cells) were seeded in a 96‐well plate, incubated in the presence of BrdU with the inhibitor or vehicle for 5 days, and subjected to the BrdU ELISA assay. (C) Intracellular content of ATP. Values were calculated as ATP content per single cell, and are represented as relative to those detected in control cells. (D) The mitochondrial membrane potential was evaluated by staining with 20 nmol/L TMRM. The fluorescence intensity of cells was analyzed using a flow cytometer. After subtraction of the fluorescence intensity of nonstained cells, the median fluorescent signal values were calculated. (E) Morphology of mitochondria. After treatment with vehicle (left) or ML364 (right), the cells were stained with MitoTracker Red CMXRos. Scale bars represent 10 µm. (F) Mitochondrial accumulation of ROS. For the last 30 min of incubation, the cells were treated with the mitochondrial ROS indicator MitoSOX Red. Scale bars represent 20 µm. Nuclei were stained with Hoechst33342. Data are expressed as means ± one standard deviation of six (A‐C) or seven (D) wells of a representative experiment of three experimental series.*P < 0.05 versus vehicle‐treated cells (B‐D). *P‐*values obtained from Student’s *t*‐test are also shown (A). Microscopic images are representative of three experiments (E, F).

Results are presented as means ± one standard deviation. Comparison between groups was performed with a Student’s *t*‐test (for Figs. 1F‐G, 2D, 3, 4B, 5, 6, 7A‐D), or one‐way (for Fig. 1D) or two‐way ANOVA (Figs. 1E and 2A) using Kaleida Graph (Hulinks, Tokyo, Japan). For post hoc testing of ANOVA, we conducted Turkey’s test. Statistical significance at *P* < 0.05 is indicated with an asterisk in each graph. In cases with *P *> 0.05, the *P* value is shown in the graphs.

## Results

### *Usp2* deficiency repressed proliferation and differentiation in C2C12 myoblasts

To assess the roles of USP2 in myoblasts, we first generated *Usp2*KO myoblasts using the CRISPR/Cas9 system. We designed the gRNA targeting isopeptidase‐coding region of the *Usp2* gene and obtained three independent clones. Clone#1 had an aberrant *Usp2* gene coding internal deletion (resulting in loss of amino acid residues 280–289) in an allele and a frame shift corresponding to C‐terminal amino acid residue 335 in the other allele (Fig. 1A). Similarly, clone#2 also had an internal deletion (resulting in loss of amino acid residues 273–289) and frame shift (corresponding to C‐terminal amino acid residue 335) in the *Usp2* gene. In contrast, clone#3 had a frame shift in both alleles of the *Usp2* gene, resulting in mutation of C‐terminal amino acid residues 347 and 341. Putative products encoded by all *Usp2* variants exhibited mutations in the ubiquitin protease domain.

We conducted RT‐PCR analysis using primers specifically designed for the mutated region to confirm genome editing by the CRISPR/Cas9 system. cDNA of the *Usp2*KO cell lines yielded lower amounts of amplification products versus the control C2C12 cells (Fig. 1B). Thus, mRNA transcribed from the large deleted allele of each *Usp2*KO cell line lacked the sequences recognized by the PCR primers. In addition, we investigated the levels of USP2 protein in control C2C12 and *Usp2*KO cells using commercially available antibodies. The results showed that the expression of the protein was low in C2C12 cells (data not shown).

Park et al. (2002) previously reported that rat myoblast‐like L6 cells displayed defects of differentiation after the transfection of a dominant negative form of USP2. In agreement with this report, *Usp2*KO C2C12 cells did not express the myosin heavy chain even after culture in the differentiation medium for 7 days (Fig. 1C). Similarly, a 7‐day culture in the differentiation medium did not increase the *Myog* transcript in any of the *Usp2*KO clones (Fig. 1D). Thus, USP2 is involved in the differentiation of both mouse and rat myoblast cell lines. Since the three C2C12‐derived *Usp2*KO cell lines showed principally similar transcriptome profiles (data not shown), we in the following show results obtained from clone#2.

We next assessed the effects of *Usp2* deficiency on proliferation. As shown in Fig. 1E, accumulation of MTT‐derived formazan was significantly repressed in *Usp2*KO C2C12 cells compared to control C2C12 cells after incubation for 3 and 5 days. Additionally, incorporation of BrdU was markedly decreased in *Usp2*KO cells compared to control cells in growth condition for 5 days (Fig. 1F). It can, therefore, be concluded that depletion of USP2 decreases proliferation of myoblasts.

Myoblasts have the potential to migrate into the damaged region (Yin et al., 2013). Thus, migration of myoblasts is thought to participate in tissue regeneration in pathological as well as physiological situations. We, therefore, also compared the motility of *Usp2*KO cells and control cells. A total of 823 ± 131 C2C12 cells were present in the unseeded region of the well after 2 days of incubation in growth medium (Fig. 1G). Similarly, 952 ± 236 *Usp2*KO cells (1.16 ± 0.29‐fold compared to control cells) moved from the seeded region under an identical culture condition. These results indicate that USP2 is dispensable for the migration of myoblasts under normal culture conditions.

### *Usp2* deficiency suppressed oxidative phosphorylation in C2C12 myoblasts

Insufficient supply of glucose is known to repress proliferation and differentiation but not migration of C2C12 cells (Topman et al., 2012; Elkalaf et al., 2013). Therefore, we considered the possible involvement of USP2 in glucose‐derived ATP production. To evaluate this possibility, we measured the intracellular content of ATP in *Usp2*KO cells and control C2C12 cells under normal or glucose‐free culture conditions. As shown in Fig. 2A, the chemiluminescent signal correlating to ATP content decreased by ~25% in *Usp2*KO cells compared to control cells, indicating an aberrant production of ATP in *Usp2*KO cells. On the other hand, the intracellular level of ATP was not significantly different in *Usp2*KO cells versus control cells after incubation in glucose‐free DMEM for 12 h, although the level tended to be lower in the former cells.

A previous study demonstrated that proliferation of myoblasts is highly dependent on OXPHOS by the mitochondrial respiratory chain (Pääsuke et al., 2016). We, therefore, analyzed oxygen consumption by ESR. We measured the line widths of ESR spectra (Fig. 2B) and calculated the *p*O_2_ every 3 min (Fig. 2C). Subsequently, the oxygen consumption rate (OCR) was obtained as a slope of the regression line of *p*O_2_ (Fig. 2D). The OCR of C2C12 cells was ~ 6mmHg/min under normal culture conditions. In contrast, *Usp2*KO cells demonstrated a ~50% decrement in OCR. Since the OCR is mainly determined by OXPHOS, USP2 deficiency seems to inhibit ATP synthesis in the mitochondria of cultured myoblasts.

### *Usp2* deficiency did not affect mitochondrial content in C2C12 myoblasts

Since repression of oxygen consumption in *Usp2*KO myoblasts might be attributed to a decrement of mitochondria in myoblasts, we measured the mitochondrial content in *Usp2*KO cells and control cells. Fig. 3A represents the relative copy number of mtDNA determined by the content of D‐loop elements. Although the D‐loop content tended to decrease in *Usp2*KO cells versus control cells, the difference was not statistically significant (0.86‐fold, *P* = 0.457, n = 3; Fig. 3A). Moreover, the total mass of MitoTracker Red CMXRos‐stained mitochondria was not affected by *Usp2* deficiency in myoblasts (*P* = 0.898, n = 11‐13; Fig. 3B). These results collectively indicate that USP2 does not modify the mitochondrial content in myoblasts.

### *Usp2* deficiency affects the morphology of mitochondria in C2C12 cells

In addition to the number of mitochondria, the activity of respiratory chain enzymes is another determinant of oxygen consumption. The morphology of the mitochondria reflects their activity: low‐activity and damaged mitochondria are fragmented (fission), whereas high‐activity mitochondria are fused followed by elongation (Liesa and Shirihai, 2013; Ikeda et al., 2015). Thus, we monitored the MitoTracker Red CMXRos‐stained mitochondria of *Usp2*KO and control C2C12 cells (Fig. 4A). In control cells, the mitochondria were filamentous, branched, and connected to other mitochondria forming mesh networks. In contrast, *Usp2*KO cells showed granular mitochondria mainly around the nucleus. The proportion of network‐forming mitochondria was ~ 45% and 7% in control cells and *Usp2*KO cells, respectively (Fig. 4B). We also stained mitochondria using another mitochondria staining chemical, MitoTracker Green FM (Thermo Fisher Scientific). As shown in Fig. 4C, this staining yielded similar results: fragmented mitochondria were abundantly observed in *Usp2*KO cells compared with control cells. Moreover, transfection of mitochondria‐targeting mCherry showed that filamentous mitochondria were dominant in control C2C12 cells. In contrast, most mitochondria in *Usp2*KO cells were fragmented (Fig. 4D). Collectively, USP2 contributes to maintain a high‐activity mitochondrial network.

### *Usp2* deficiency did not modulate the activities of respiratory chain complexes in C2C12 cells

Since *Usp2*KO cells manifested a malfunction of mitochondria, we measured the intrinsic enzymatic activities of the respiratory chain complexes in vitro using a frozen‐thawed mitochondrial fraction. In the respiratory chain, complexes I, III, and IV produced proton gradients between the intermembrane space and matrix of the mitochondrium. As shown in Fig. 5A‐C, the activities of complexes I, III, and IV did not show differences between *Usp2*KO cells and control cells (*P* = 0.663, 0.523, and 0.990, respectively; n = 5). On the other hand, *Usp2* deficiency in C2C12 cells also had negligible (*P* = 0.708, n = 5) impact on the enzymatic activity of the proton gradient‐driven ATPase, also called complex V (Fig. 5D). Hence, USP2 does not seem to directly control mitochondrial complexes.

### *Usp2* deficiency affects the mitochondrial membrane potential, membrane permeability, and production of mitochondrial ROS

Since *Usp2* deficiency did not modify the activities of the respiratory chain complexes, we considered that a proton leak might occur in *Usp2*KO cells. To assess this possibility, we monitored the membrane potential using depolarization‐sensitive TMRM. Incubation (30 min) of C2C12 cells with 20 nmol/L TMRM produced an intense TMRM signal (Fig. 6A). In contrast, median signal intensity was slightly but significantly decreased in *Usp2*KO cells. It can thus be concluded that USP2 maintains the mitochondrial membrane potential by inhibition of a proton leak.

We next treated *Usp2*KO cells and control cells with calcein‐AM and cobalt chloride. In control C2C12 cells, an intense calcein‐AM‐derived green fluorescent signal was detected (Fig. 6B). Since the cobalt ion – a quencher of the calcein signal – did not enter mitochondria in control C2C12 cells, membrane permeability is maintained at a low level in the cells. In contrast, the signal intensity of calcein‐AM was significantly decreased in *Usp2*KO cells, indicating that mitochondrial membrane permeability was increased in these cells.

Mitochondrial membrane permeability and potential are regulated by the local production of ROS (Sanderson et al., 2013; Yu et al., 2019). We, therefore, next examined the production of ROS in the mitochondria of *Usp2*KO cells and control C2C12 cells. The median value of the MitoSox Red‐derived signal—a ROS indicator—was>12‐fold higher in *Usp2*KO cells than in control cells after incubation for 30 min (Fig. 6C). Therefore, *Usp2* deficiency accumulates mitochondrial ROS in C2C12 cells.

### Chemical inhibition of USP2 influences proliferation, intracellular levels of ATP, mitochondria integrity, and accumulation of mitochondrial ROS in C2C12 cells

We assessed the effects of a chemical inhibitor of USP2 on C2C12 cells to confirm the hypothesis that the observed defects in *Usp2*KO cells are attributed to inhibition of USP2. Treatment with the USP2‐selective inhibitor ML364 (10 µmol/L) did not release LDH from C2C12 cells for the first 12 h after application (Fig. 7A). Moreover, ML364 did not significantly increase the release of LDH at 16 h and 24 h (data not shown). These findings indicate that this dose was not toxic for C2C12 cells. Treatment with this dose of ML364 for 5 days caused > 70% decrease in the incorporation of BrdU to the cells; thus, ML364 decreased the proliferation of C2C12 cells (Fig. 7B). On the other hand, treatment with ML364 for 8 h also decreased the intracellular level of ATP to 64.9% of vehicle‐treated cells (Fig. 7C). Moreover, treatment with ML364 markedly decreased the potential of the mitochondrial membrane in C2C12 cells (0.37‐fold difference vs. that of vehicle‐treated cells, *P* < 0.001; Fig. 7D). Furthermore, granular mitochondria were present in ML364‐treated cells, whereas most vehicle‐treated cells contained filamentous mitochondria (Fig. 7E). Fig. 7G shows the time course of changes in the accumulation of mitochondrial ROS, which was visualized with MitoSOX Red, after treatment with ML364. After 30 min of treatment, the intensity of the fluorescent signal was higher in ML364‐treated cells versus vehicle‐treated cells. Accumulation of ROS was also evident 2 h after addition of ML364. Therefore, treatment with ML364 promoted the accumulation of ROS in the mitochondria of C2C12 cells, as well as a decrement in mitochondria integrity, intracellular level of ATP, and proliferation.

## Discussion

In this study, we observed a significant decrement in intracellular ATP in *Usp2*KO C2C12 cells cultured in glucose‐containing DMEM. In contrast, there was no difference in ATP content between control and *Usp2*KO cells cultured under the glucose‐free condition. DMEM contains other nutrients (e.g., pyruvate and glycogenic amino acids) (Yao and Asayama, 2017), which are utilized in the tricarboxylic acid (TCA) cycle (Katz, 1985). Given that OXPHOS is closely linked to the TCA cycle, we initially considered that USP2 deficiency may interrupt the accumulation of ATP even under the glucose‐free condition. However, impairment of ATP accumulation was not evident in *Usp2*KO cells cultured under the glucose‐free condition, with both control and *Usp2*KO cells showing low levels of ATP. Thus, the production of ATP in C2C12 cells may be highly dependent on the presence of glucose. To support this, a previous study demonstrated that C2C12 cells exhibited lower maximal respiration and drastic inhibition of proliferation under glucose‐free conditions (Elkalaf et al., 2013). In any case, USP2 may contribute to maintaining the supply of ATP in C2C12 cells in the presence of glucose.

Myoblasts and satellite cells are thought to mainly utilize glycolysis for ATP supply, whereas myotubes obtain ATP by OXPHOS (Wagatsuma and Sakuma, 2013; Sin et al., 2016). However, emerging evidence indicates that the role of OXPHOS in myoblasts is not negligible. For instance, myoblasts require OXPHOS for proliferation and differentiation, suggesting a critical role of the mitochondrial respiratory chain in myoblasts (Deepa et al., 2016). Moreover, proliferating muscle progenitor cells exhibited a metabolic switch from fatty acid and pyruvate oxidation to glycolysis during early proliferation, and the mitochondrial content and gene expression of the TCA cycle were increased at a later period (Ryall et al., 2013). In the present study, we demonstrated that USP2 contributes to the generation of sufficient ATP by OXPHOS in myoblasts. On the other hand, *Usp2*KO cells exhibited impairments in proliferation and differentiation, both of which require an adequate glucose‐derived energy supply by the mitochondria (Elkalaf et al., 2013; Riddle et al., 2018). Collectively, these results suggest that USP2 might control proliferation and differentiation in myoblasts through sustained activation of mitochondria.

Mitochondria drastically change their morphology depending on their activation status (Liesa and Shirihai, 2013). For instance, mitochondria damaged by oxidative stress undergo fission and subsequently fragmentation (2014). It has been proposed that mitochondrial fission and fragmentation are parts of the process of mitophagy, removing aberrant mitochondria from cells (Yu and Pekkurnaz, 2018). A previous study demonstrated that adiponectin represses H_2_O_2_‐induced mitophagy in C2C12 cells (Ren et al., 2017). On the other hand, adiponectin induces USP2 in mammary epithelial cells (Treeck et al., 2008). In addition, the present study demonstrated that lack of USP2 caused mitochondria fission. Collectively, these observations indicate that USP2 might prevent mitophagy‐triggering malfunction in the mitochondrium.

Although this study did not clarify the substrates of USP2 in myoblasts, *Usp2* deficiency severely disturbed the generation of the mitochondrial membrane potential. Since USP2 negligibly affected the activities of complexes I, III, IV, and V, loss of the mitochondrial membrane potential is attributable to proton leak rather than modulation of respiratory chain activity. One of the possible mechanisms underlying proton leak is driven by ROS (Navet et al., 2006). *Usp2*KO cells exhibited a higher mitochondrial ROS accumulation than control cells. ROS were locally produced by mitochondrial complexes I and III following an imbalance in the enzymatic activities of the mitochondrial complexes (Jastroch et al., 2010; Lenaz et al., 2010). Although our study did not provide direct evidence, several previous studies have indicated the consequences of these events. For example, mitochondrial ROS decreased membrane potential and OXPHOS activity (Sanderson et al., 2013; Wen and Garg, 2018). Moreover, ROS have the potential to accelerate membrane permeability, causing a decrease in the potential of the mitochondrial membrane through enhanced movement of H^+^ and K^+^ to the inner membrane (Shanmughapriya et al., 2015; Yu et al., 2018). On the other hand, the membrane potential is a crucial factor of ATP production in the electron transport system of mitochondria (Brand and Nicholls, 2011). Collectively, these findings suggest that USP2 deficiency decreases the potential of the mitochondrial membrane via the production of ROS, resulting in a decrement of ATP production. Thus far, studies have not determined the direct molecular target(s) of USP2, which may be responsible for the accumulation of ROS induced by USP2 deficiency. Interestingly, in a preliminary study, we found that the levels of uncoupling protein 2, a major mitochondrial protein involved in the removal of ROS in myocytes (Jun et al., 2015; Gerö and Szabo, 2016; Pan et al., 2018), were decreased in *Usp2*KO C2C12 cells. Further studies are required to uncover detailed molecular events in USP2‐controlled ROS production and mitochondria integrity in myoblasts.

In addition to the genetic deletion of USP2 by genome editing, we evaluated the effects of ML364 on C2C12 cells. A previous study demonstrated that ML364 attenuated cell cycle progression in Mino and HCT116 cancerous cells by promoting the degradation of cyclin D1 (Davis et al., 2016). Thus, we can not exclude the possibility that ML364 inhibited the proliferation of C2C12 cells independently of the ATP supply. Nevertheless, the nontoxic dose of ML364 altered the mitochondrial morphology and membrane potential, enforcing the effects of USP2 in mitochondria integrity. Notably, the accumulation of ROS in mitochondria was apparent in C2C12 cells following treatment with ML364, even if this was carried out for only a short period. The genetic and chemical blockade of USP2 causes accumulation of ROS in mitochondria, and impedes the potential of the mitochondrial membrane. Therefore, USP2 appears to play crucial roles in the integrity of mitochondria for the efficient supply of ATP.

## Conflict of interest

All authors declare that they have no competing interests.
